# Ultrahigh-performance vector magnetic field sensor with wedge-shaped fiber tip based on surface plasmon resonance and magnetic fluid

**DOI:** 10.1515/nanoph-2022-0224

**Published:** 2022-07-12

**Authors:** Zijian Hao, Yongxi Li, Shengli Pu, Jia Wang, Fan Chen, Mahieddine Lahoubi

**Affiliations:** College of Science, University of Shanghai for Science and Technology, 200093, Shanghai, China; Shanghai Key Laboratory of Modern Optical System, University of Shanghai for Science and Technology, 200093, Shanghai, China; Photonlabs Instruments Inc., Shanghai, China; Department of Physics, Laboratory L.P.S., Badji-Mokhtar Annaba University, 23000, Annaba, Algeria

**Keywords:** magnetic field, magnetic fluid, reflection-type, sensor probe, surface plasmon resonance

## Abstract

A novel fiber-optic vector magnetic field sensor and its sensing quality dependent of fabrication method has been proposed and investigated. The proposed sensor has two surfaces on the tip of a multimode fiber, which is used as the sensing probe. By plating different thickness of gold film on the surfaces, surface plasmon resonance (SPR) can be generated and the signal can be reflected by the surfaces as well. Meanwhile, magnetic fluid (MF) as the magnetic field sensitive material is packed around the sensing probe. The experimental results prove that the response of MF to external magnetic field can be used to sense magnetic field intensity and direction via monitoring the dip wavelength of SPR. The obtained refractive index (RI) sensitivities are 2105 nm/RIU (RI range: 1.332–1.365) and 6692 nm/RIU (RI range: 1.372–1.411), magnetic field intensity sensitivities are 11.67 nm/mT (0°), and −0.47 nm/mT (90°). Besides, the proposed sensing probe is ultracompact and the footprint is extremely small (the length of sensing part is only 615 μm), which is very helpful for magnetic field detection in narrow space and gradient field.

## 1 Introduction

Magnetic fluid (MF) is a kind of nanomaterial, which is stable colloid composed of surfactant-coated magnetic nanoparticles (MNPs) and carrier liquid. MNPs rotate and interact with each other under certain magnetic field, which leads to the magneto-induced local refractive index (RI) change [1–7]. Since MF combining with optical fiber were proposed to measure magnetic field [8], many MF-based optical fiber magnetic field sensors using different sensing structures and principles were developed, e.g. MF-filled Fabry–Perot interferometer [9], fiber surface plasmon resonance (SPR) [10, 11], MF-coated optical fiber multimode interferometer [12–18].

Recently, much attention about MF-based fiber-optic magnetic field sensor has been paid to spectral response to magnetic field direction, which is the fundamental for vector magnetic field sensing [10]. It mainly relies on the non-central symmetric fiber structure.

However, most of the reported MF-based fiber-optic magnetic field fiber sensors are classified as *transmission-type*, which is based on measuring the transmission spectrum. Thus, they are not conducive to sense the magnetic field in narrow space. In 2020, Gao et al. proposed an elliptical core micro-FBG to measure the reflected light [19], but its RI sensitivity (156 nm/RIU) still needs to be improved, which is due to the insensitivity of FBG structure to the change of external RI.

SPR as a measurement technique can detect the tiny RI change over metal film surface by strong evanescent field [20–28], which provides a new-fashioned approach to realize high-sensitivity magnetic field sensing. In 2015, Liu. et al. proposed a SPR sensor with a twin-core fiber tip. The high-sensitivity RI measurement is realized (6463 nm/RIU) [29]. Such sensing scheme enables the smaller size of the device and is suitable for sensing in very narrow space. However, the applied twin-core fiber has a very small size (3.8 μm core diameter), which requires high processing accuracy. Meanwhile, its symmetrical geometry could not realize vector sensing. It is also complex for coupling the individual signals in-and-out of dual-core or multi-core fiber [30, 31].

In this work, a *reflection-type* vector magnetic field sensing probe is proposed. It composes of a gold-plated wedge-shaped multimode fiber (MMF) tip. MF is utilized to coat the sensing probe. It can measure both the magnetic field intensity and direction. Such structure has the following three main advantages: (1) First and most importantly, the sensor is ultracompact. The length of the sensing part is only 615 μm. As the spatial resolution is inversely proportional to the length of the sensing area, hyperspatial resolution is obtained. Therefore, such sensor has higher accuracy for gradient magnetic field measurement. (2) Besides, comparing with single mode fiber (SMF), the 105 μm-core MMF employed in this work is easy to generate SPR with higher fabrication robustness. (3) Furthermore, the fiber with larger core diameter allows light propagation with higher power for the incident halogen lamp source, which will increase the signal to noise ratio (SNR) [32]. It also has high magnetic field sensitivity. The proposed sensor is a typical example for realizing *lab-on-a-fiber* [33].

## 2 Results and discussion

**Figure 1:**
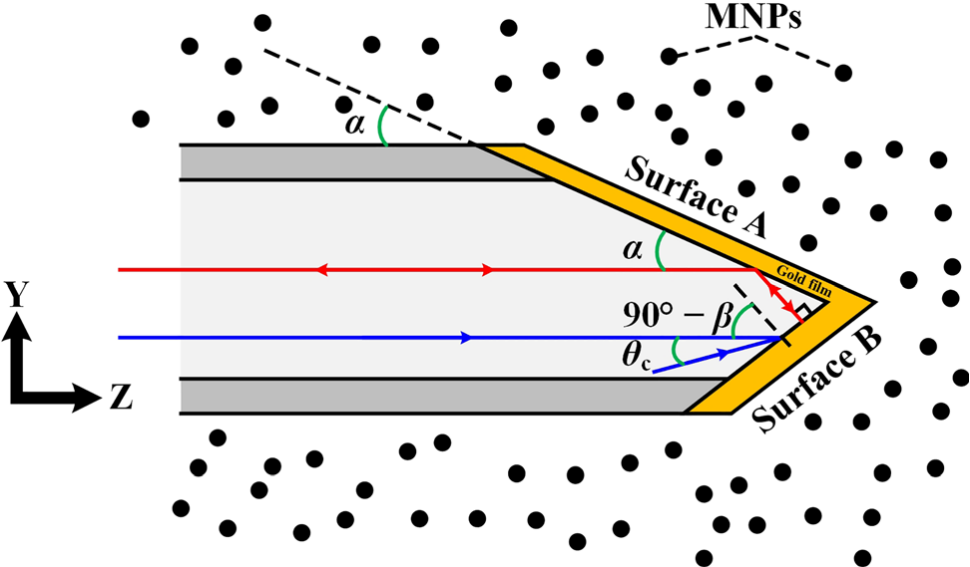
Sensing structure of the wedge-shaped SPR multimode fiber sensing probe.

The schematic diagram of the wedge-shaped sensing probe is shown in Figure 1. It is fabricated by grinding and polishing the MMF. The sensing surface (referred as Surface A) with grinded angle *α* and reflective surface (referred as Surface B) with grinded angle *β* are coated with gold films of different thickness (where 2*α* + *β* = 90°). Surface A and gold film constitute the Kretschmann configuration [20, 34]. The incident light will excite SPR at Surface A. When the light is perpendicularly reflected on Surface B, it will be reflected back to Surface A and excites SPR again. But the SPR cannot be excited on Surface B. The SPR propagation constant *β*_sp_ and resonance angle *θ*_sp_ can be expressed as [21].

(1) Reβsp=k0Reεmεsεm+εs=k0nco⁡sinθsp,

(2) θsp=arcsinReεmεsεm+εs/nco≈84°,

where *k*_0_ and *ε*_m_ are free-space wave number and dielectric constant of gold film. *ε*_s_ and *n*_co_ are the dielectric constant of surrounding medium and fiber core refractive index (at *λ* = 680 nm, *ε*_m_ = −15.051 + 1.0516i, *ε*_s_ ≈ 1.84, *n*_co_ = 1.455). The incident cut-off angle *θ*_c_ of the employed MMF (NA = 0.23) is ∼13.3° [35, 36]. As shown in Figure 1, the incident angle to Surface B is *θ*_iB_ = 90° − *β* + *θ*_c_ << 84°, which cannot meet the prerequisite for exciting SPR.

It should be pointed out that the incident light on the sensing surface stems from two factors. One is the light directly incident on the sensing surface. Its incident angle is 90° − *α*. The other is the light coming from the reflective surface. Its incident angle is 90° − 2*α*.

**Figure 2:**
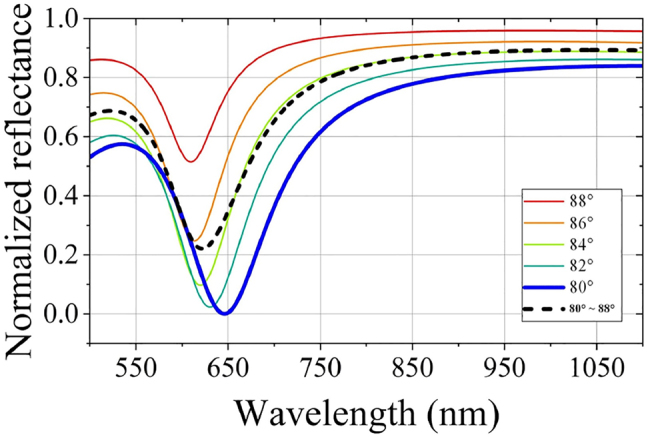
Calculated SPR reflectance spectra at different incident angles.

To analyze the possible influence of multimode excitation on the sensing performance, Figure 2 shows the calculated SPR reflectance spectra at different incident angles. The SPR reflectance spectrum contributed to the multiple incident angles (superimposing the reflectance spectra corresponding to all incident angles) is indicated as the black dot line. It is obvious that the FWHM of attenuation peak is not significantly broadened in the incident angle ranging from 80° to 90°.

**Figure 3:**
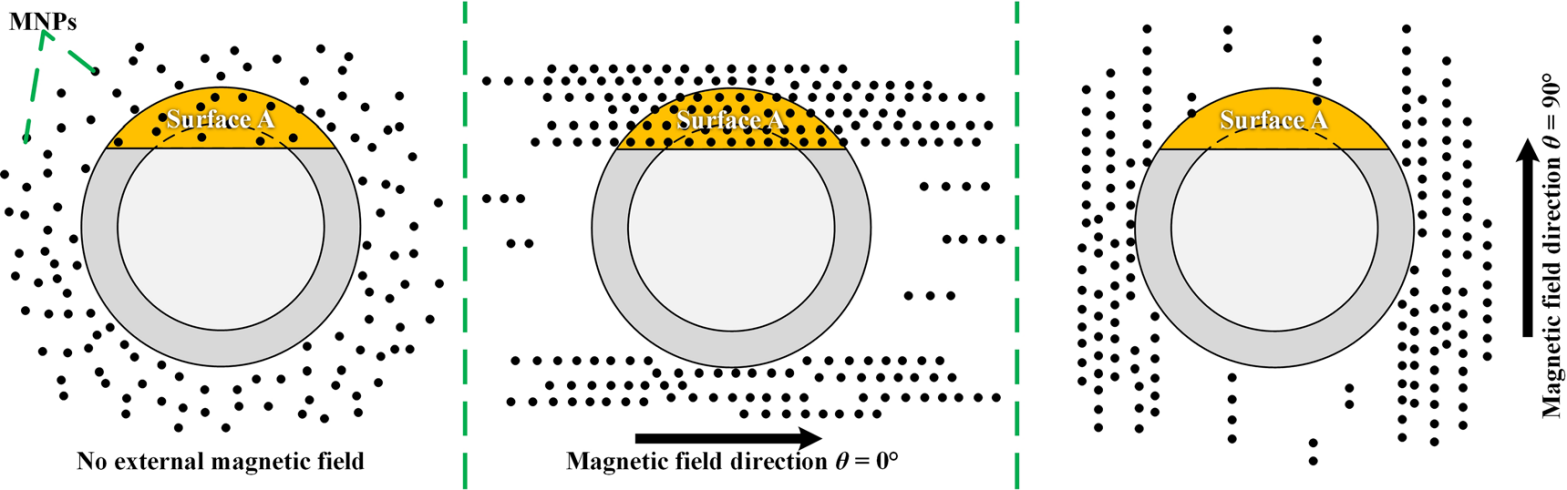
Distribution of MNPs under different magnetic field direction.

A series of experiments have proved that the MNPs in MF will rotate and interact with each other under external magnetic field [11, 14, 15]. The MNPs will form nanochain-clusters around fiber and then the local RI will be changed (see Figure 3), which is dependent on the external magnetic field intensity and direction. As a result, the resonance wavelength of SPR will be modulated by both intensity and direction of the external magnetic field, which is the basic principle for vector magnetic field sensing.

**Figure 4:**
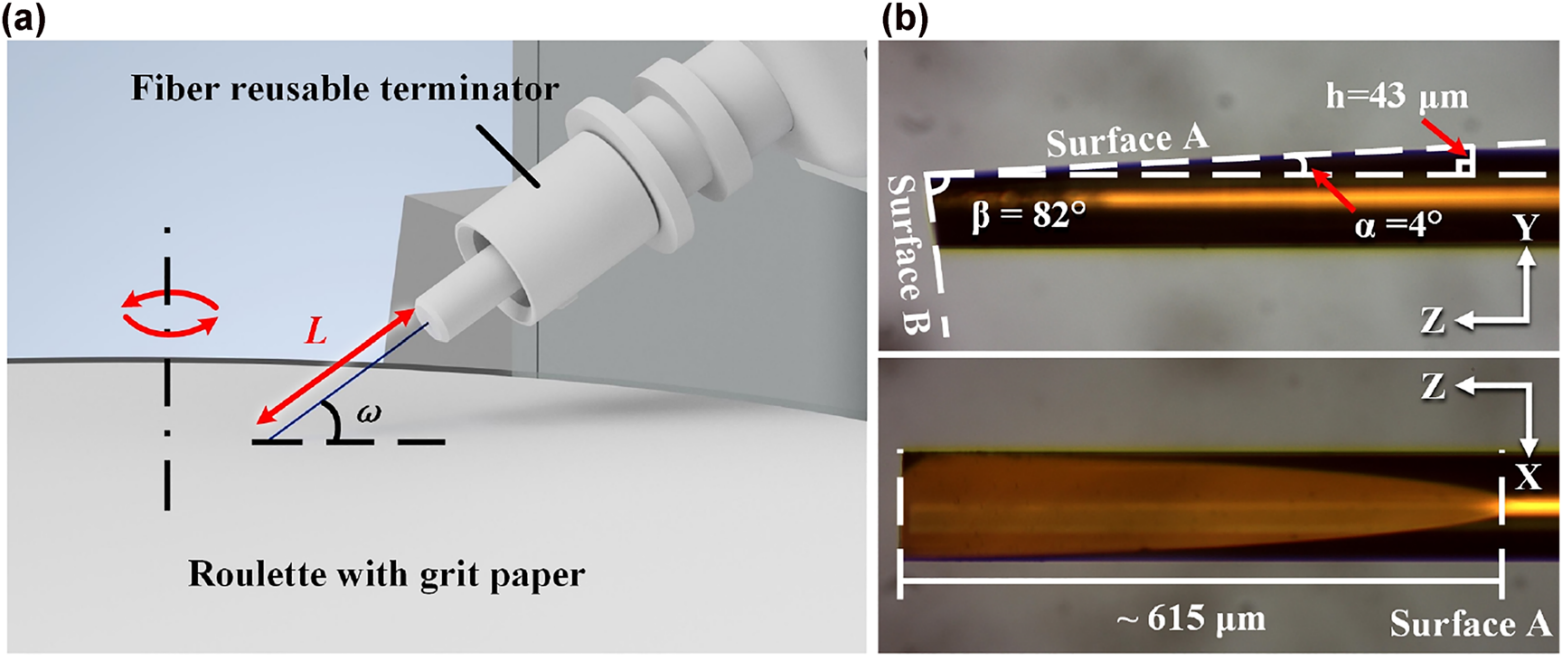
Grinding method and micrographs of the sample: (a) Partially enlarged view of the grinding and polishing system. The red double-arrow straight line and red circular arrow indicate the length of the fiber to be grinded and the rotation direction of the roulette, respectively; (b) micrographs of the wedge-shaped fiber tip after plating gold film. The white dotted lines are guided for eyes.

A home-made optical fiber end-face processing system has been employed for fabricating the wedge-shaped fiber tip. As shown in Figure 4a, the MMF is fixed in a fiber reusable terminator at a certain grinding angle and is then grinded by roulette with grit paper. A high-speed brushless motor with about 153 rad/s rotating speed is set to drive the roulette. 2000-grit, 4000-grit, 8000-grit and 10,000-grit paper are employed to grind and Polish the fiber surface, respectively.

**Figure 5:**
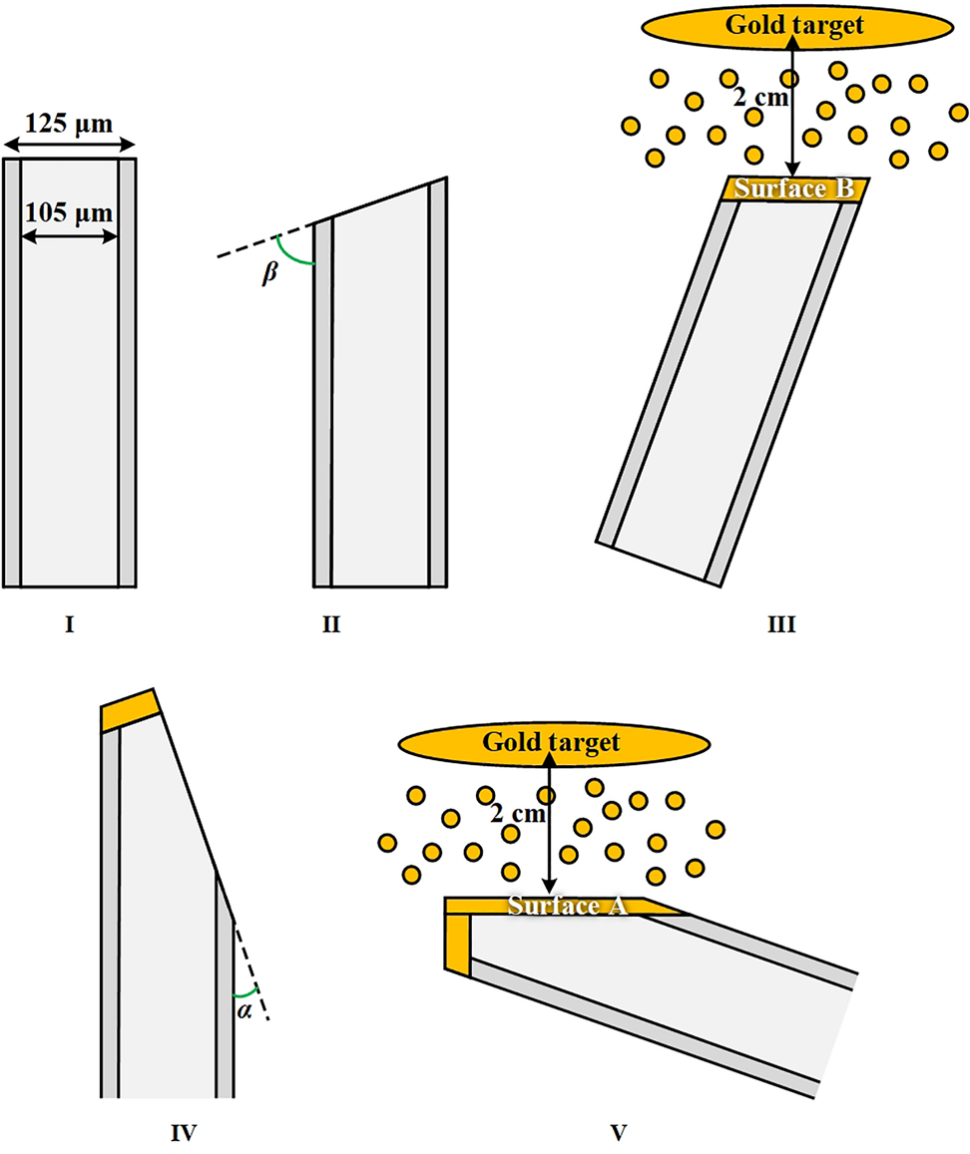
Flowchart for fabricating the wedge-shaped fiber tip sensor probe based on SPR.

Specifically, four main steps are required to fabricate the designed wedge-shaped fiber tip (see the flowchart shown in Figure 5): (1) Grinding and polishing Surface B with tilt angle *β*; (2) Regulating Surface B and making it parallel the ion source, and then plating it with gold film with sputtering current of 10 mA and sputtering duration larger than 180 s; (3) Grinding and polishing Surface A with tilt angle *α*; (4) Regulating Surface A and make it parallel the ion source, and then plating it with gold film with sputtering current of 10 mA and sputtering duration of 180 s. Through this “two-step” deposition method, Surface A and B can be deposited with gold film of different thicknesses. Surface B with relatively thick gold film is only used as the reflector (the corresponding polishing angle *β* is out of the SPR excitation range), while Surface A with relatively thin gold film acts as the sensing surface. Thus, the thickness of gold film on Surface A will definitely and solely affect the sensing performance. The micrographs of the as-fabricated sensor probe are shown in Figure 4b.

**Figure 6:**
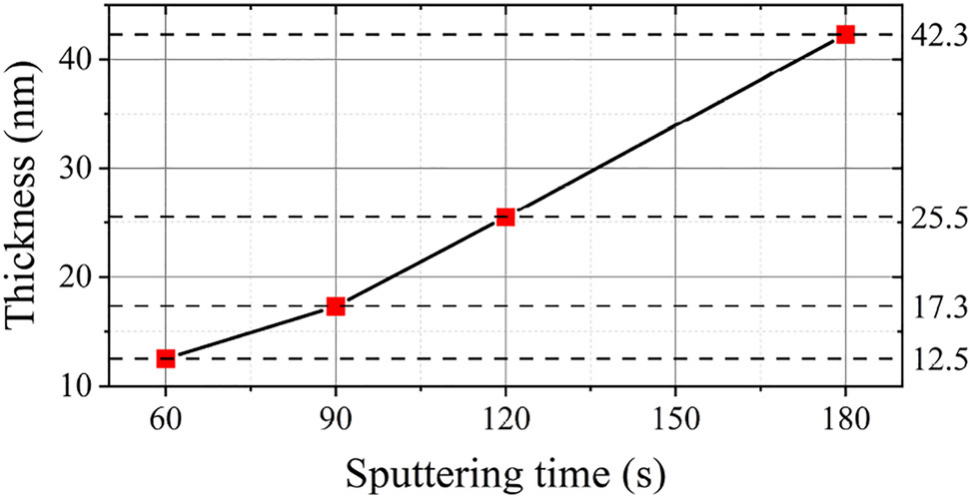
Relationship between gold film thickness and sputtering duration.

The thickness of the gold film on Surface A is about 42.3 nm, which is measured with an atomic force microscope (Dimention Icon, Bruker). The measured gold film thickness as a function of sputtering time is shown in Figure 6.

**Figure 7:**
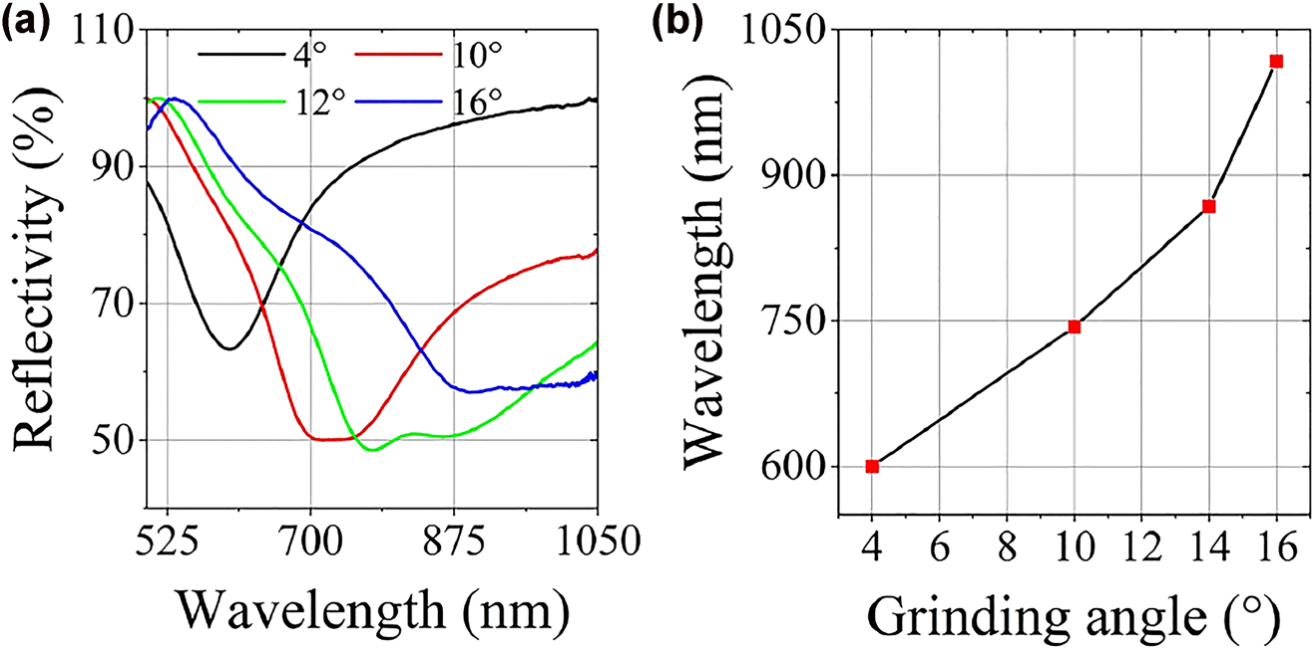
Sensor performance for the sensing surface with different grinding angels: 4°, 10°, 12°, 16°. The analyte is water with RI of 1.332: (a) SPR spectra; (b) resonance wavelengths.

**Figure 8:**
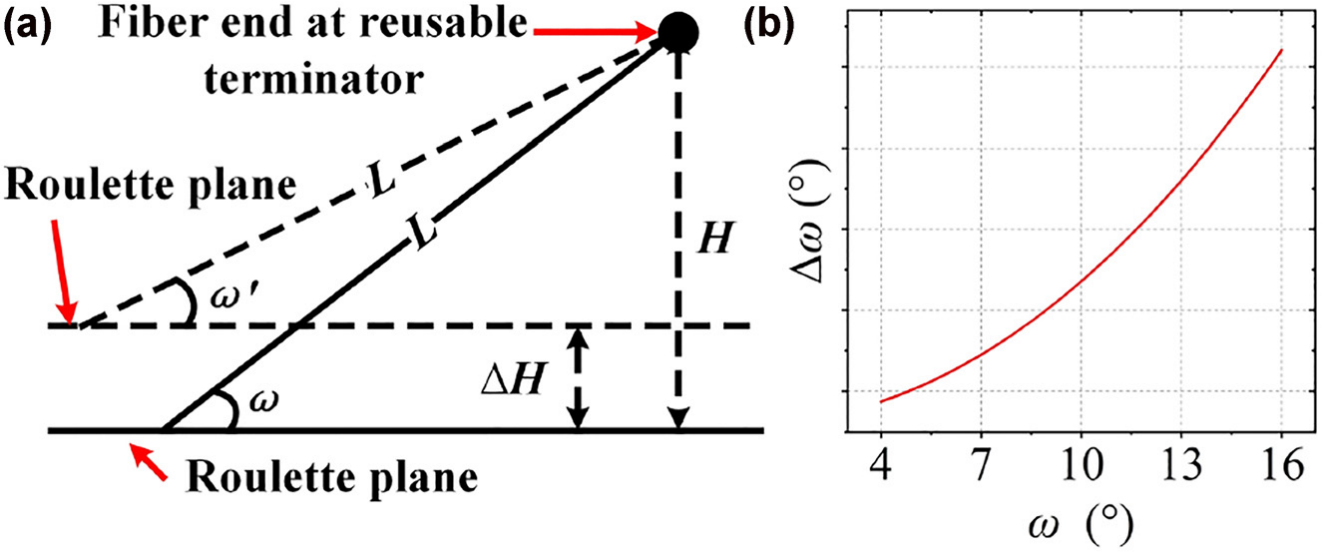
Model for the machining error analysis and the corresponding results: (a) Model for analyzing the influence of machining error. The horizontal solid and dashed lines are the relative positions of the fiber and the roulette before and after the roulette plane is raised. The oblique lines represent the fiber; (b) Relationship between grinding angle error *Δω* and grinding angle *ω*.

Influence of grinding angle on the SPR spectrum has been investigated to select the appropriate processing parameters. As the grinding angle increases, the resonance dip redshifts and splits (see Figure 7). Machining error occurs due to up and down jittering caused by uneven grinding roulette of the optical fiber end-face processing system. Then, two surfaces with different grinding angle will be formed, which accounts for the splitting of resonance dip. To analyze the impact of machining error, the theoretical model is established in Figure 8a. Considering the fixed and relatively short (∼5 mm) length of the fiber sticking out of the fiber reusable terminator *L* (see Figures 4a and 8a), the extruded optical fiber can be considered as a rigid body [37]. So, the rise-up of the roulette plane will lead to the fiber bending upward. Then, *H* will change to *H* − ∆*H*. Thus, the grinding angle will change from *ω* to *ω′*. ∆*H* is the jittering amplitude of the roulette; *ω* and *ω*′ are grinding angles before and after roulette plane rising up. Therefore, the grinding angle error can be expressed as:

(3) $$\Delta \omega =\omega -\omega^{\prime }=\omega -\arcsin\left(\sin\left(\omega \right)-\frac{\Delta H}{L}\right).$$

Obviously, the machining error ∆*ω* increases with *ω*. The numeral result is plotted in Figure 8b. Thereby, a sensor sample with *α* = 4°, *β* = 82° and *h* = 43 μm (see Figure 4b) is employed to avoid machining error impact.

**Figure 9:**
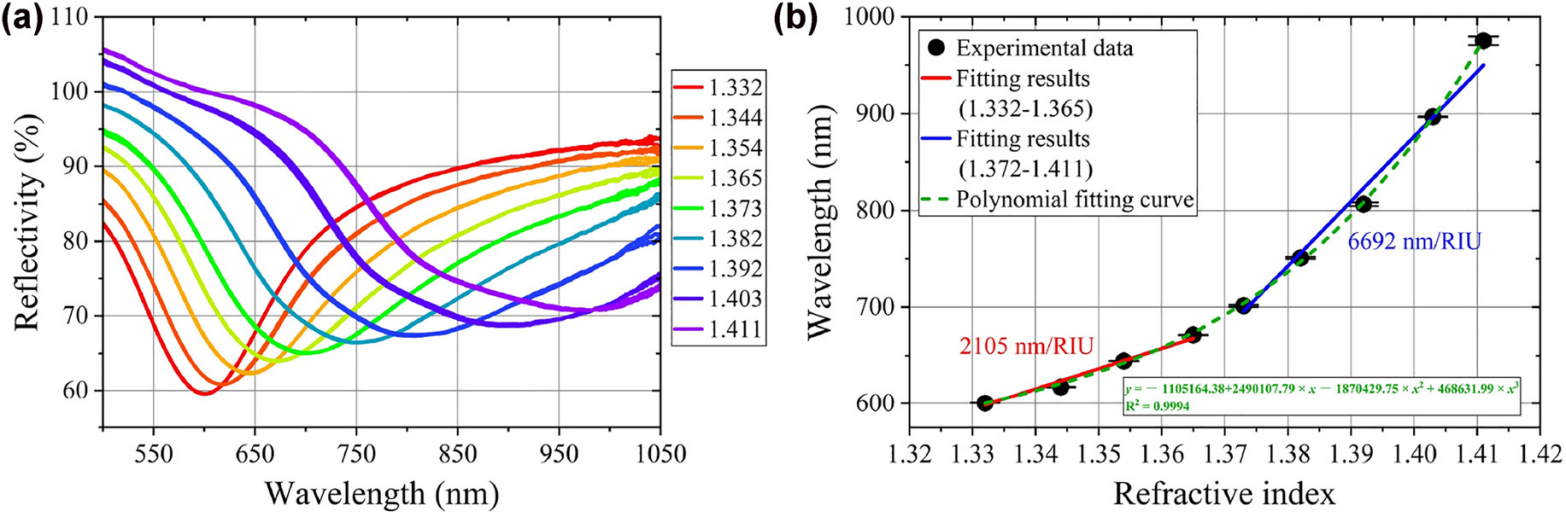
RI response of the as-fabricated sensing probe: (a) SPR spectrum response to different RIs; (b) relationship between resonance wavelength and RI.

Figure 9 shows the SPR spectra for the sensing probe immersed in glycerol solution with different RIs (1.332–1.411) and the corresponding fitting curve of resonance wavelength with RI. In order to facilitate the application of sensing, the linear fitting in two limited variation ranges is employed. The obtained sensitivities are 2105 and 6692 nm/RIU, respectively. Each RI case has been tested for three times. The good robustness is obtained (the first three repeated measurement show very good consistency and in order to guarantee the quality of the gold film and then the accuracy of measurement, the limited times of repeated measurement is employed). In order to make the magneto-induced RI change of MF in the linear response range of the SPR dip wavelength, the MF with RI of 1.354 is employed.

**Figure 10:**
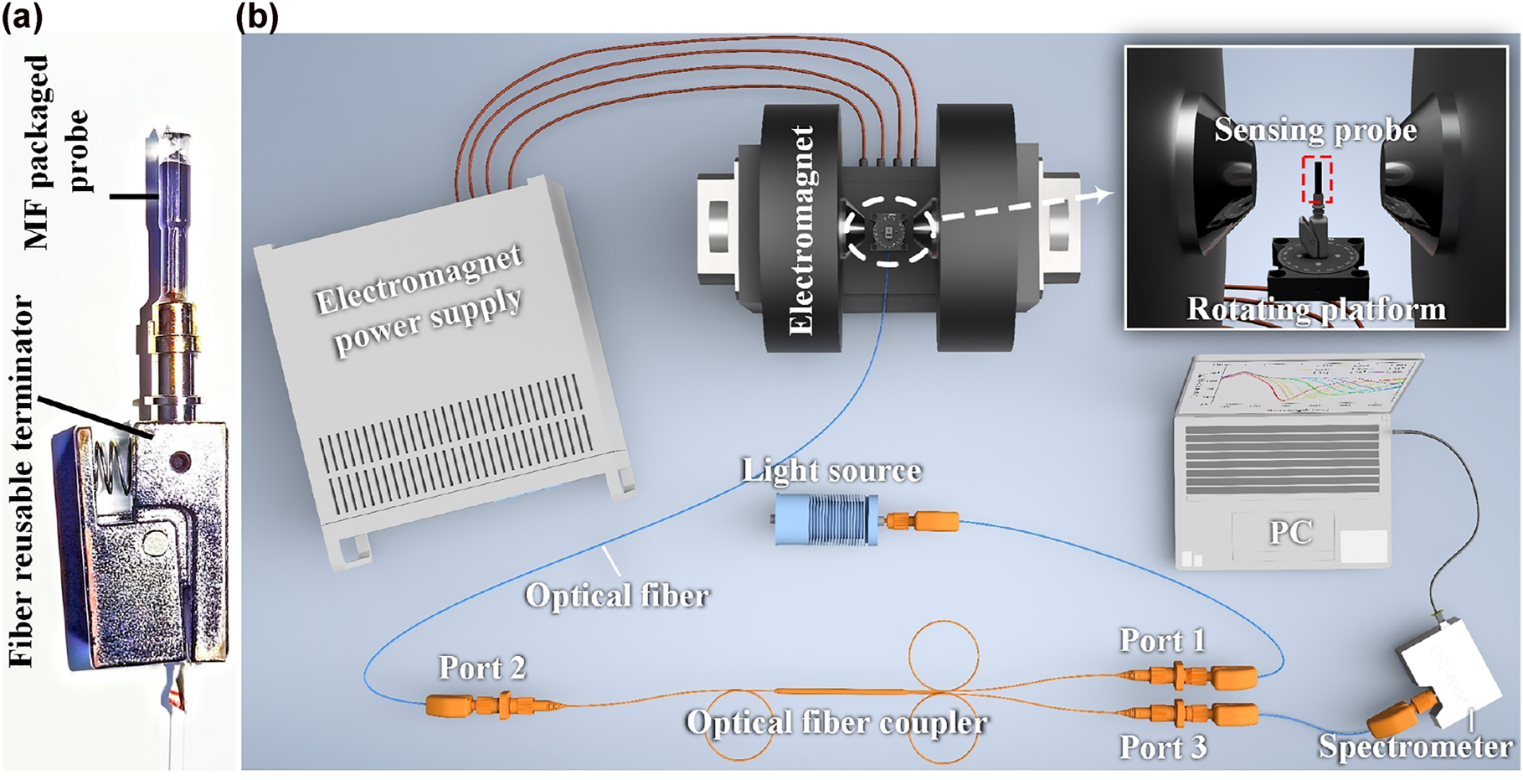
Photograph of sensor and schematic diagram of the experimental setup: (a) Photograph of the as-fabricated sensing probe. The wedge-shaped sensing tip is placed in a plastic tube with MF and then sealed with UV glue and fixed at the top of the “fiber reusable terminator”; (b) Diagram of the experimental setup for investigating the sensing properties.

As for the device packaging, the gold-plated wedge-shaped fiber tip is fixed in a fiber reusable terminator. Plastic pipe and UV glue are used to package the sensing probe with MF (EMG605, FerroTec) (see Figure 10a). The size of the packaged fiber probe is about 2 cm long, but the sensor performance is only dependent on the RI near the sensing surface (surface A). Thereby, the whole size of the packaged fiber probe is only determined by the packaging technology, which can be improved considerably without degrading the sensor performance. This implies that the packaging-dependent volume of the MF will not affect the sensing performance notably. But the concentration of the employed MF will affect its’ RI, and then change the FWHM of the reflectance spectrum and the magnetic field sensitivity of the sensor. Figure 10b shows the experimental setup for investigating the sensing properties. A tungsten halogen light source (HL-2000, Ocean Insight) with spectrum range of 360–2400 nm is used. The incident light comes from Port 1 of the fiber coupler and is reflected by the sensing probe through Port 2. Finally, it is received by the optical spectrum analyzer (USB4000, Ocean Insight) through Port 3. A power supply (KEITHLEY 2260A-30-72, Tektronix) is employed to control the external magnetic field intensity. A rotating platform is used to adjust *θ* (see Figure 3 for the definition of *θ*).

**Figure 11:**
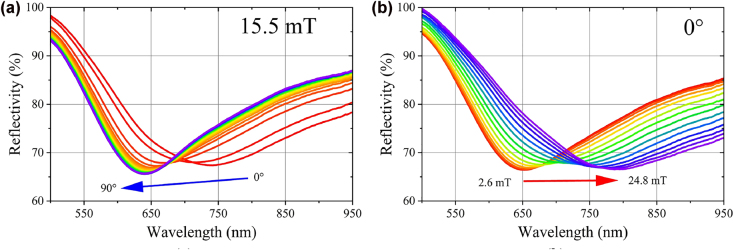
Reflectance spectrum for the as-fabricated sensing probe at different magnetic field direction (*B* = 15.5 mT) (a) and different magnetic field intensities (*θ* = 0°) (b).

**Figure 12:**
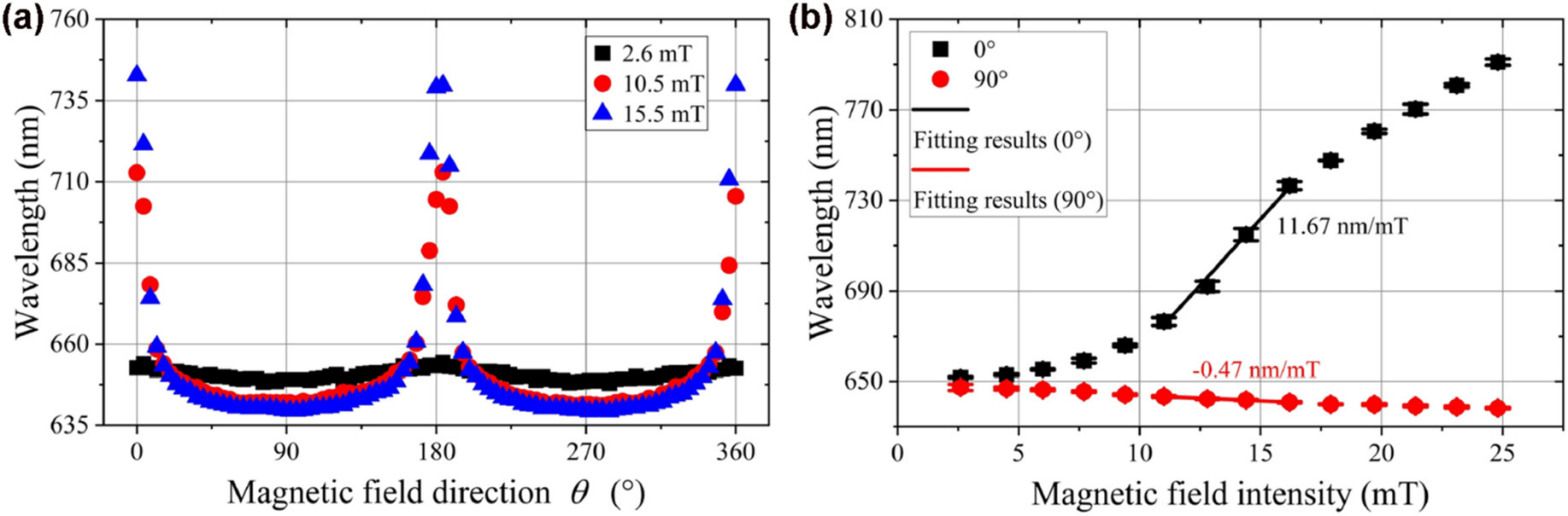
SPR dip wavelength as a function of external magnetic field direction (*θ*) at different intensities (a) and external magnetic field intensity (*B*) at 0° and 90° direction (b).

The reflectance spectrum for the as-fabricated sensing probe at different magnetic field direction *θ* and intensities *B* are measured at room temperature. The typical results are shown in Figure 11a (*B* is fixed at 15 mT) and 10 b (*θ* is fixed at 0°), respectively. To be explicit, the corresponding SPR dip wavelength of the as-fabricated sensing probe at different magnetic field direction and intensities is extracted and plotted in Figure 12. Similar response curves are obtained at *B* = 2.6, 10.5 and 15.5 mT. Maximum and minimum response occurs at 0°/180° and 90°/270° (see Figure 12a), respectively. As shown in Figure 12b, the SPR dip wavelength redshifts and blueshifts with the increase of magnetic field intensity at 0° and 90°, respectively. The achieved sensitivity of the as-fabricated magnetic field sensor is 11.67 nm/mT (0°) and −0.47 nm/mT (90°). For the 0.02 nm resolution of the spectrometer (HR4000, Ocean Insight) employed in our experiments, the detection limit is 1.7 μT (0°) and 42.6 μT (90°). However, the detection limit is critically dependent on the resolution of the employed spectrometer/interrogator. Considering the 1 pm resolution of the typical commercial interrogator (e.g. SI720, Micron Optics, Inc.), the lowest detection limit is 0.85 μT (0°) and 2.12 μT (90°). The experimental results show that the proposed sensing probe can be used to detect magnetic field intensity and direction.

In addition, the refractive index of MF is also sensitive to temperature. So, temperature effect and compensation are worth investigating. It may be an easy and simple way to address this issue through cascading a fiber Bragg grating. However, the length of the grating is generally in several centimeters. Cascading the proposed sensor with a fiber Bragg grating will greatly reduce the sensor integration, which is not conducive for miniaturization.

Usually, the actual magnetic field is gradient in space. If the magnetic field gradient is relatively large within a relatively small space, the detecting result should be the average value. Thereby, it is necessary to consider the spatial resolution. It is convenient to refer to the figure of merit (FOM) to compare the sensor performance comprehensively. Herein, the FOM is defined as the magnetic intensity sensitivity divided by the spatial resolution (minimum detectable space size) and expressed as

(4) $$FOM=S_{\text{m}}/R_{\text{s}},$$

where *S*_m_ and *R*_s_ indicates the magnetic field intensity sensitivity and spatial resolution, respectively.

Performance indicators of relevant sensing configurations are presented in Table 1. The previous works based on D-shaped, U-bent and off-set spliced fiber structures without SPR [15,40–46] are simple to fabricate, but have limited sensitivity. Microfiber-based sensors have the advantage of high sensitivity [38, 47, 48]. Especially, the magnetic field intensity sensitivity of our recently reported microfiber coupler structure can reach 97.86 nm/mT theoretically [36], but the mechanical strength is relatively low. The D-shaped fiber and titled fiber Bragg grating combined with SPR can achieve high sensitivity and good mechanical strength, but the length of D-shaped fiber structure or fiber grating is usually in several centimeters (limited by their processing technology), which will result in a relatively poor spatial resolution. Magnetic field sensors based on Fabry–Perot resonators may have high sensing performance, but the “vector” function is disabled due to the symmetrical configuration. By contrast, the *reflection-type* SPR-based sensing scheme proposed in this work not only has **high sensitivity** (11.67 nm/mT) but also has a significant advantage in spatial resolution, which is one to two orders of magnitude superior to those of other structures. The obtained FOM is as high as 18975.61 mT^−1^, which is around **200–5020 times higher** than those of the ordinary sensors (non-SPR and non-microfiber type).

**Table 1:** Sensing performance of related fiber-optic magnetic field sensors.

Sensing structure	Type	Sensitivity (nm/mT)	Spatial resolution (m)	FOM (mT^−1^)	Vector sensing	Ref.
D-shaped fiber + SPR	Transmissive	6.92	∼6.00 × 10^−3^	∼1153.56	Yes	[39]
D-shaped SNS	Transmissive	2.37	2.50 × 10^−2^	94.80	Yes	[40]
D-shaped SMS	Transmissive	0.53	∼8.00 × 10^−3^	∼66.25	Yes	[41]
U-bent SMF	Transmissive	0.52	∼1.00 × 10^−2^	∼52.00	Yes	[42]
U-bent SMS	Transmissive	3.19	5.00 × 10^−2^	63.80	No	[43]
Core-offset SMF	Transmissive	0.41	1.50 × 10^−2^	27.35	No	[44]
Core-offset SMF	Transmissive	0.17	2.50 × 10^−2^	6.80	Yes	[45]
Core-offset PCF	Transmissive	0.115	∼3.04 × 10^−2^	∼3.78	Yes	[15]
Lateral-offset TCF	Transmissive	0.22	1.97 × 10^−2^	11.18	Yes	[46]
TFBG + SPR	Transmissive	1.80	∼1.00 × 10^−2^	∼180.00	Yes	[11]
Microfiber coupler	Transmissive	1.31	2.50 × 10^−2^	52.40	Yes	[47]
Microfiber coupler	Transmissive	97.86	6.50 × 10^−3^	15050.87	No	[38]
Tapered + SPR	Transmissive	10.00	∼6.34 × 10^−3^	∼1577.29	No	[48]
Fabry–Perot	Reflective	0.40	3.6 × 10^−5^	11111.11	No	[49]
Fabry–Perot	Reflective	0.33	7 × 10^−5^	4714.29	No	[50]
Fabry-Perot + Vernier	Reflective	10.26	7.95 × 10^−5^	1324120.75	No	[51]
**Wedge-shaped + SPR**	**Reflective**	**11.67**	**∼6.15 × 10** ^−**4**^	**18975.61**	**Yes**	**This work**

The bold values means to emphasize that this is the work of this paper.

Finally, our preliminary experiments show that the optical fiber surface roughness affects the FWHM of the reflectance spectrum and resonant wavelength. The details of this will be investigated in future work with the ultracompact multichannel sensor. Moreover, the size ratio of the sensing surface to the reflective surface needs to optimize to maximize both the reflective signal and the corresponding extinction ratio, and then improve the sensing performance further.

## 3 Conclusions

In conclusion, an ultracompact vector magnetometer based on SPR and MF is proposed and experimentally demonstrated. The MMF with 105 μm core diameter is employed, whose large core diameter can accept reflection angle with larger machining error. The sensing and reflective surfaces have no special requirements for grinding depth, which makes the fabrication process easier. The incident optical power can be higher with the MMF probe, which will increase the SNR. The *high sensitivity* and *hyperspatial resolution* are achieved. It is believed that this structure can provide a novel scheme for designing fiber-optic vector magnetic field sensing based on MF and enable the realization of *lab-on-a-fiber*.
